# Metabolomics profiling of xenobiotics in elite athletes: relevance to supplement consumption

**DOI:** 10.1186/s12970-018-0254-7

**Published:** 2018-09-27

**Authors:** Fatima Al-Khelaifi, Ilhame Diboun, Francesco Donati, Francesco Botrè, Mohammed Alsayrafi, Costas Georgakopoulos, Noha A. Yousri, Karsten Suhre, Mohamed A. Elrayess

**Affiliations:** 1 Anti Doping Laboratory Qatar, ADLQ, Sports City, P.O Box 27775, Doha, Qatar; 2 UCL-Medical School, Royal Free Campus, NW3 2PF, London, UK; 3 Department of Economics, Mathematics and Statistics, Birkbeck, University of London, WC1E 7HX, London, UK; 4 Laboratorio Antidoping, Federazione Medico Sportiva Italiana, Largo Giulio Onesti 1, 00197 Rome, Italy; 5 Department of Genetic Medicine, Weill Cornell Medical College in Qatar, Qatar-Foundation, P.O. Box 24144, Doha, Qatar; 6 Department of Physiology and Biophysics, Weill Cornell Medical College in Qatar, Qatar-Foundation, P.O. Box 24144, Doha, Qatar

**Keywords:** Metabolomics, Xenobiotics, Sport, Athletes, Nutritional supplements

## Abstract

**Background:**

Supplements are widely used among elite athletes to maintain health and improve performance. Despite multiple studies investigating use of dietary supplements by athletes, a comprehensive profiling of serum supplement metabolites in elite athletes is still lacking. This study aims to analyze the presence of various xenobiotics in serum samples from elite athletes of different sports, focusing on metabolites that potentially originate from nutritional supplements.

**Methods:**

Profiling of xenobiotics in serum samples from 478 elite athletes from different sports (football, athletics, cycling, rugby, swimming, boxing and rowing) was performed using non-targeted metabolomics-based mass spectroscopy combined with ultrahigh-performance liquid chromatography. Multivariate analysis was performed using orthogonal partial least squares discriminant analysis. Differences in metabolic levels among different sport groups were identified by univariate linear models.

**Results:**

Out of the 102 detected xenobiotics, 21 were significantly different among sport groups including metabolites that potentially prolong exercise tolerance (caffeic acid), carry a nootropic effect (2-pyrrolidinone), exert a potent anti-oxidant effect (eugenol, ferulic acid 4 sulfate, thioproline, retinol), or originate from drugs for different types of injuries (ectoine, quinate). Using Gaussian graphical modelling, a metabolic network that links various sport group-associated xenobiotics was constructed to further understand their metabolic pathways.

**Conclusions:**

This pilot data provides evidence that athletes from different sports exhibit a distinct xenobiotic profile that may reflect their drug/supplement use, diet and exposure to various chemicals. Because of limitation in the study design, replication studies are warranted to confirm results in independent data sets, aiming ultimately for better assessment of dietary supplement use by athletes.

## Background

Dietary supplements are widely used by athletes in different sports as they promise improved competitive performance [1]. Supplements include vitamins, minerals, protein, creatine, and various “ergogenic” compounds [2]. Several studies studied supplement use by elite athletes based on interviews and surveys. An investigation of the nutritional supplements in 1620 Norwegian elite athletes revealed higher use of nutritional supplements in male elite athletes than controls, with coaches being the main advisors for types and quantity of supplements used [3]. Data obtained from doping control forms of 361 Danish elite athletes revealed 100% use one or more nutritional supplement [4]. A survey of dietary supplements among 164 elite young German athletes revealed 80% used at least one supplement including minerals, vitamins and energy drinks. Carbohydrates were most frequently consumed, whereas only a minority reported use of protein/amino acids, creatine, or other ergogenic aids [5].

Previous studies, however, have also suggested that most athletes are ill-informed about the benefits and risks of supplements, due, at least in part, to the limited scientific evidence available [2]. Since elite athletes are usually unwilling to take part in trials testing the effectiveness of supplements due to their intense training and competition schedules, such evidence remains limited. Therefore, a retrospective approach that examines the presence of potential dietary supplements and their metabolites in elite athletes may provide valuable insight into what had previously been consumed by elite athletes and its predicted impact on their health and performance.

Metabolomics presents a comprehensive approach for detecting metabolic changes in response to dietary, lifestyle, and environmental factors [6]. Non-targeted and targeted metabolomics-based approaches have significantly improved the simultaneous profiling of hundreds of metabolites underlying various metabolic pathways [7]. Despite recommendations of comprehensive metabolic analyses in human nutrition to ensure that all aspects of health are accurately assessed [8], its utilization remains limited [9].

Although multiple studies have investigated the consumption of specific dietary supplements by elite athletes through interviews and surveys, no study has adopted a retrospective comprehensive serum xenobiotic profiling. This study, therefore, aims to analyze differences in various xenobiotics in serum samples from elite athletes of different sports, focusing on metabolites that potentially originate from supplement/drug consumption.

## Methods

### Materials

Authentic standards of d7-glucose, d3-leucine, d8-phenylalanine and d5-tryptophan were purchased from Cambridge Isotope Laboratories (Andover, MA). D5-hippuric acid, d5-indole acetic acid and d9-progesterone were procured from C/D/N Isotopes, Inc. (Pointe-Claire, Quebec). Bromophenylalanine was provided by Sigma-Aldrich Co. LLC. (St. Louis, MO) and amitriptyline was from MP Biomedicals, LLC. (Aurora, OH). Recovery standards of DL-2-fluorophenylglycine and DL-4-chlorophenylalanine were from Aldrich Chemical Co. (Milwaukee, WI). Tridecanoic acid was purchased from Sigma-Aldrich (St. Louis, MO) and d6-cholesterol was from Cambridge Isotope Laboratories (Andover, MA). Standards for the HILIC dilution series of alpha-ketoglutarate, ATP, malic acid, NADH and oxaloacetic acid were purchased from Sigma-Aldrich Co. LLC. (St. Louis, MO) while succinic acid, pyruvic acid and NAD+ were purchased from MP Biomedicals, LLC. (Santa Ana, CA).

### Study design

Study participants included in this study were 478 elite athletes (411 males and 67 females) from different sports (315 football, 44 athletics, 35 cycling, 16 rugby, 38 swimming, 17 boxing and 13 rowing) who participated in national or international sports events and tested negative for doping substances at anti-doping laboratories in Qatar and Italy. Ethnicities included 85% Europeans, 10% Americans and 5% Africans. There was no evidence of population stratification in sport groups based on ethnicity. Spare serum samples, collected by doping control officers for anti-doping human growth hormone tests, were used for metabolomics studies as described previously [10]. This study was performed in accordance with the World Medical Association Declaration of Helsinki. All protocols were approved by the Institutional Research Board of anti-doping lab Qatar (F2014000009). Accordingly, only information related to sport type, gender and ethnic group were available for researchers. Table 1 summarizes number, gender, prevalence and ethnicities of participants as per study groups.

**Table 1:** Characterization of study participants

Sport group	Number (Gender)	Prevalence %	Ethnicity
Athletics	44 (22 M, 22F)	9	97.8% EU, 2.3% AF
Boxing	17 (1 M, 16F)	4	100% EU
Cycling	35 (31 M, 4F)	7	100% EU
Football (soccer)	315 (315 M)	66	80% EU, 13% AM, 7% AF
Rowing	13 (6 M, 7F)	3	100% EU
Rugby	16 (16 M)	3	87.5% EU, 12.5% AM
Swimming	38 (20 M, 18F)	8	94.7% EU, 2.6% AM, 2.6% AF

Numbers of participants, their gender (M: males, F: females), prevalence and predicted ethnicities (EU: Europeans, AF: Africans, AM: Americans) are shown per sport groups

### Metabolomics

Metabolomics profiling was performed using established protocols at Metabolon, Durham, NC, USA. All methods utilized a Waters ACQUITY ultra-performance liquid chromatography (UPLC) and a Thermo Scientific Q-Exactive high resolution/accurate mass spectrometer interfaced with a heated electrospray ionization (HESI-II) source and Orbitrap mass analyzer operated at 35,000 mass resolution.

### Sample preparation

Following receipt, samples were inventoried and immediately stored at -80 °C until processed. Samples were prepared using the automated MicroLab STAR® system from Hamilton Company. Several recovery standards were added prior to the first step in the extraction process for quality control (QC) purposes. To remove protein, dissociate small molecules bound to protein or trapped in the precipitated protein matrix, and to recover chemically diverse metabolites, proteins were precipitated with methanol under vigorous shaking for 2 min (Glen Mills GenoGrinder 2000) followed by centrifugation. The resulting extract was divided into five fractions: two for analysis by two separate reverse phase (RP)/UPLC-MS/MS methods with positive ion mode electrospray ionization (ESI), one for analysis by RP/UPLC-MS/MS with negative ion mode ESI, one for analysis by HILIC/UPLC-MS/MS with negative ion mode ESI, and one sample was reserved for backup. Samples were placed briefly on a TurboVap® (Zymark) to remove the organic solvent. The sample extracts were stored overnight under nitrogen before preparation for analysis.

### Quality control (QC)

Several types of controls were analyzed in concert with the experimental samples. These included a pooled matrix sample generated by taking a small volume of each experimental sample (or alternatively, use of a pool of well-characterized human plasma) served as a technical replicate throughout the data set. Extracted water samples served as process blanks. A cocktail of QC standards that were carefully chosen not to interfere with the measurement of endogenous compounds were spiked into every analyzed sample, allowed instrument performance monitoring and aided chromatographic alignment. Instrument variability was determined by calculating the median relative standard deviation (RSD) for the standards that were added to each sample prior to injection into the mass spectrometers. Overall process variability was determined by calculating the median RSD for all endogenous metabolites (i.e., non-instrument standards) present in 100% of the pooled matrix samples. Experimental samples were randomized across the platform run with QC samples spaced evenly among the injections.

### Ultrahigh performance liquid chromatography-tandem mass spectroscopy (UPLC-MS/MS)

The sample extract was dried then reconstituted in solvents compatible to each of the four methods. Each reconstitution solvent contained a series of standards at fixed concentrations to ensure injection and chromatographic consistency. One aliquot was analyzed using acidic positive ion conditions, chromatographically optimized for more hydrophilic compounds. In this method, the extract was gradient eluted from a C18 column (Waters UPLC BEH C18–2.1 × 100 mm, 1.7 μm) using water and methanol, containing 0.05% perfluoropentanoic acid (PFPA) and 0.1% formic acid (FA). Another aliquot was also analyzed using acidic positive ion conditions, however it was chromatographically optimized for more hydrophobic compounds. In this method, the extract was gradient eluted from the same afore mentioned C18 column using methanol, acetonitrile, water, 0.05% PFPA and 0.01% FA and was operated at an overall higher organic content. Another aliquot was analyzed using basic negative ion optimized conditions using a separate dedicated C18 column. The basic extracts were gradient eluted from the column using methanol and water, however with 6.5 mM Ammonium Bicarbonate at pH 8. The fourth aliquot was analyzed via negative ionization following elution from a HILIC column (Waters UPLC BEH Amide 2.1 × 150 mm, 1.7 μm) using a gradient consisting of water and acetonitrile with 10 mM Ammonium Formate, pH 10.8. The MS analysis alternated between MS and data-dependent MS/MS scans using dynamic exclusion. The scan range varied slighted between methods but covered 70–1000 m/z. Raw data files are archived and extracted as described below.

### Data extraction and compound identification

Raw data was extracted, peak-identified and QC processed using Metabolon’s hardware and software. These systems are built on a web-service platform utilizing Microsoft’s. NET technologies, which run on high-performance application servers and fiber-channel storage arrays in clusters to provide active failover and load-balancing. Compounds were identified by comparison to library entries of purified standards or recurrent unknown entities. Metabolon maintains a library based on authenticated standards that contains the retention time/index (RI), mass to charge ratio (*m/z)*, and chromatographic data (including MS/MS spectral data) on all molecules present in the library. Furthermore, biochemical identifications are based on three criteria: the correct retention time/index to the authentic standard, the correct m/z within 10 ppm of the authentic standard and the correct fragmentation spectrum (MS/MS) to the standard. The MS/MS scores are based on a comparison of the ions present in the experimental spectrum to the ions present in the library spectrum. While there may be similarities between these molecules based on one of these factors, the use of all three data points can be utilized to distinguish and differentiate biochemicals. More than 3300 commercially available purified standard compounds have been acquired and registered into Laboratory Information Management System (LIMS) for analysis on all platforms for determination of their analytical characteristics, however this study reports data on detected xenobiotics classified by Metabolon as follows: Metabolon data analysts use proprietary visualization and interpretation software to confirm the consistency of peak identification among the various samples. Library matches for each compound were checked for each sample and corrected if necessary. Metabolon classified known metabolites by “super-pathway”, representing chemical classes, and “sub-pathway”, corresponding to the specific role of a compound in metabolism, on the basis of the Kyoto Encyclopedia of Genes and Genomes (KEGG) pathways [11].

### Statistical analysis of metabolomics data

Metabolomics data were log-transformed to ensure normal distribution. Batch correction was already performed by Metabolon by rescaling each metabolite so that its median is equal to 1. Orthogonal partial least square discriminant analysis (OPLS-DA) was employed to identify components that distinguish sport groups from those that represent common metabolite signatures that are sport-independent. OPLS-DA was run using SIMCA 14 and included samples with less than 50% missing metabolite values. Linear models for association analysis were run using the R statistical package (version 2.14, www.r-project.org/). The model incorporated the sport groups as a categorical variable as well as covariates including gender and ethnicities. False discovery rate (FDR) multiple testing correction was performed to correct for multiple testing. Gaussian Graphical Models (GGMs) were used to identify correlated metabolites potentially leading to unbiased reconstruction of metabolic reactions as shown previously [12].

## Results

### Multivariate analysis of athlete metabolomics data

Non-targeted metabolomics was applied to determine exposure to xenobiotics and their metabolites in 478 elite athletes. The 102 identified xenobiotics belonged to six classes including 38% chemicals and drugs, 27% food components, 17% benzoate metabolites, 15% xanthine metabolites, 2% tobacco metabolites and 1% bacterial and fungal metabolites as per Metabolon’s assigned sub-pathways.

**Fig. 1:**
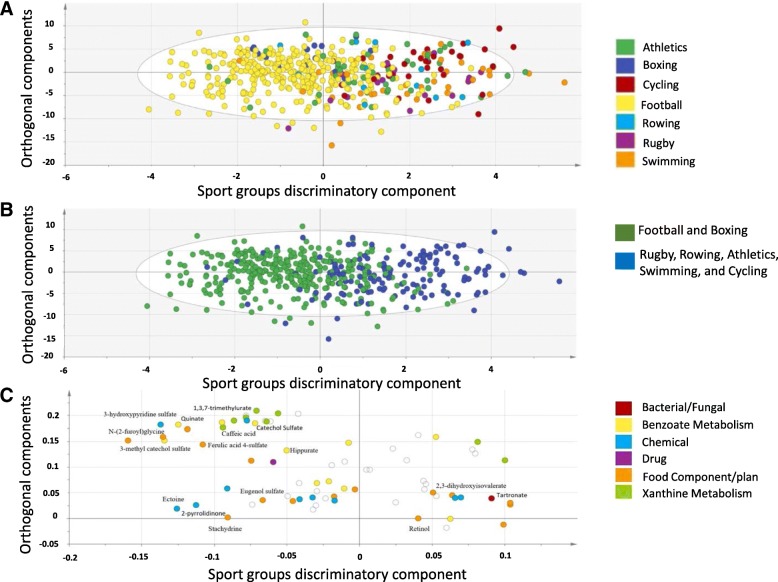
OPLS-DA model comparing elite athletes belonging to different groups of sports (athletics, boxing, cycling, football, rowing, rugby and swimming). **a** A score plot showing the class-discriminatory component (x-axis) versus orthogonal component (y-axis) among all sport groups. **b** A score plot from an updated OPLS-DA model featuring combined groups (group 1: football and boxing, group 2: rugby, rowing, athletics, swimming and cycling). **c** The corresponding loading plot from the updated model showing clusters of xenobiotics at opposite sides of group one or group either ends of the discriminatory component along the x-axis

An OPLS-DA analysis comparing xenobiotics among the studied seven sports revealed one class-discriminatory component separating football and boxing from the rest of the sport groups (Fig. 1a). For simpler visualization, OPLS-DA was repeated by combining boxing and football in one group and the rest in a second group (Fig. 1b). With the second model, the unique discriminatory component (x-axis, Fig. 1b) accounted for 40% of the differences among sport groups and 30% of the variation among xenobiotics (R-squared-Y = 0.66, Q-squared = 0.45). The corresponding loading score, shown in Fig. 1c, suggested an increase in methyl glucopyranoside, 2,3-dihydroxyisovalerate, 4-allylphenol sulfate, tartronate .hydroxymalonate, caffeic acid sulfate, ectoine, ferulic acid 4 sulfate, N-2 furoyl glycine, X3-hydroxypyridine sulfate, 4-vinylguaiacol.sulfate, catechol sulfate, hippurate, O-methylcatechol sulfate, quinate, 3-methylcatechol sulfate in boxing and football, while showing an increase in thioproline, 1,3.7-trimethylurate, tartronate (hydroxymalonate) in the other groups (athletics, cycling, rowing, rowing, rugby and swimming).

### Univariate association tests

**Fig. 2:**
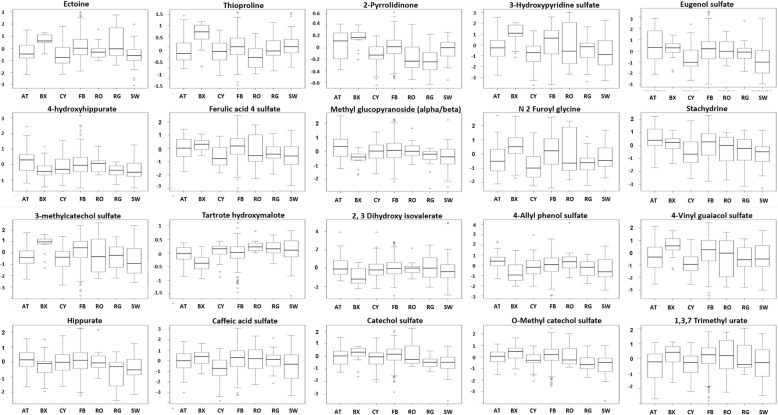
Box plots summarizing levels of significantly different metabolites among the seven studied groups (AT: Athletics, BX: Boxing, CY: Cycling, FB: Football, RO: Rowing, RG: Rugby, SW: Swimming). These levels are corrected for model’s covariates, they are mean-shifted and scaled since they represent the residuals from a repeated linear model that omits the sport group while featuring only covariates

A linear model was used to assess the significance of metabolite-associations with sport groups (athletics, boxing, cycling, football, rowing, rugby and swimming) after correcting for gender and ethnic group. Twenty one metabolites were significantly different among sport groups including benzoate metabolites (hippurate, 3-methylcatechol sulfate, 4-hydroxyhippurate), chemicals (ectoine, thioproline, 2-pyrrolidinone, 3-hydroxypyridine.sulfate), food and plant products (eugenol sulfate, ferulic acid 4 sulfate, methyl glucopyranoside (alpha/beta), 2-furoylglycine, quinate, retinol, stachydrine, tartronate (hydroxymalonate), 2,3-dihydroxyisovalerate, 4-allylphenol.sulfate, 4-vinylguaiacol.sulfate) and xanthine metabolites (caffeic acid sulfate, catechol sulfate, O-methylcatechol sulfate, and 1,3,7-trimethylurate). Table 2 summarizes the list of significantly different metabolites between different sport groups, their fold change and level of significance. Levels of significantly different metabolites in the studied 7 groups (after correction for covariates) are visually plotted in Fig. 2.

**Table 2:** Metabolites differentiating between different sport groups

Metabolite	Contrast	Fold change	Nominal *p*-value	FDR *p*-value
Catechol sulfate	Football_Swimming	0.9	3.04E-09	4.86E-06
O-methylcatechol.sulfate	Football_Swimming	1.1	3.35E-08	1.78E-05
Quinate	Football_Swimming	1.8	2.89E-08	1.78E-05
2-pyrrolidinone	Boxing_Rugby	0.5	8.13E-08	3.24E-05
2-furoyl.glycine	Cycling_Football	−1.2	6.26E-07	0.0002
2-pyrrolidinone	Boxing_Cycling	0.4	8.35E-07	0.0002
2-pyrrolidinone	Athletics_Rugby	0.3	3.99E-06	0.0009
Thioproline	Boxing_Rowing	1.2	7.42E-06	0.002
1,3,7-trimethylurate	Athletics_Football	−1.0	1.79E-05	0.003
Tartronate (hydroxymalonate)	Boxing_Rowing	−0.7	1.80E-05	0.003
2-pyrrolidinone	Football_Rugby	0.3	1.83E-05	0.003
Thioproline	Boxing_Cycling	1.0	2.96E-05	0.004
Thioproline	Athletics_Boxing	−0.8	3.50E-05	0.004
Ferulic acid 4-sulfate	Cycling_Football	−0.9	3.87E-05	0.004
Tartronate (hydroxymalonate)	Boxing_Rugby	−0.7	7.85E-05	0.007
Tartronate (hydroxymalonate)	Boxing_Swimming	−0.5	6.76E-05	0.007
3-hydroxypyridine sulfate	Cycling_Football	−1.3	7.90E-05	0.007
4-vinylguaiacol sulfate	Cycling_Football	−1.2	8.00E-05	0.007
Ectoine	Football_Swimming	0.9	8.32E-05	0.007
Ferulic acid 4-sulfate	Football_Swimming	0.9	7.10E-05	0.007
2-pyrrolidinone	Athletics_Cycling	0.2	9.09E-05	0.007
2-pyrrolidinone	Boxing_Rowing	0.4	9.30E-05	0.007
3-methyl catechol sulfate	Football_Swimming	1.1	0.0001	0.007
2,3-dihydroxyisovalerate	Boxing_Football	−1.6	0.0001	0.008
Hippurate	Football_Swimming	0.8	0.0001	0.009
Eugenol.sulfate	Athletics_Swimming	1.3	0.0002	0.009
Retinol	Athletics_Boxing	−1.50	0.0002	0.010
2,3-dihydroxyisovalerate	Athletics_Boxing	1.5	0.0002	0.010
Caffeic acid sulfate	Cycling_Football	−1.1	0.0002	0.010
Tartronate (hydroxymalonate)	Boxing_Cycling	−0.5	0.0002	0.010
3-hydroxypyridine sulfate	Football_Swimming	1.3	0.0002	0.011
Tartronate (hydroxymalonate)	Boxing_Football	−0.5	0.0002	0.013
Eugenol sulfate	Football_Swimming	1.1	0.0003	0.017
Stachydrine	Athletics_Swimming	1.1	0.0004	0.018
Ectoine	Cycling_Football	−0.8	0.0004	0.020
Stachydrine	Athletics_Cycling	1.1	0.0005	0.023
4-hydroxyhippurate	Athletics_Swimming	0.7	0.0005	0.023
Thioproline	Boxing_Football	0.8	0.0007	0.028
Methyl glucopyranoside (alpha/beta)	Athletics_Boxing	1.0	0.0008	0.031
4-allylphenol sulfate	Athletics_Boxing	1.2	0.0012	0.048

Levels of significantly different metabolites in the studied 7 groups (after correction for covariates) (FDR significance, *p* ≤ 0.05)

**Fig. 3:**
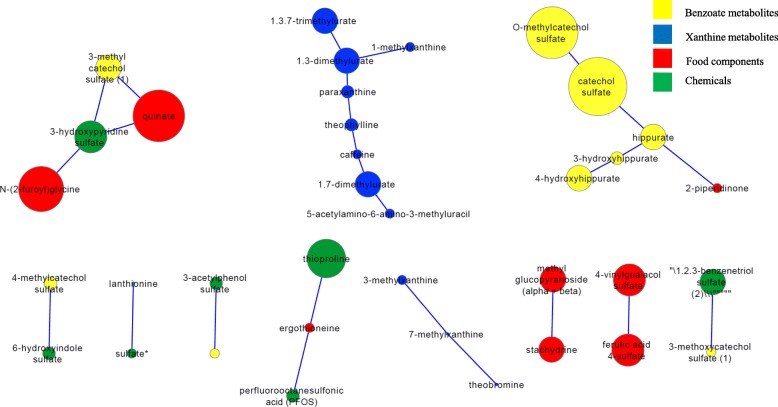
GGM sub-networks of xenobiotics that varied significantly among sport groups. Changes are represented by nodes with sizes proportional to – (log *p* value) (larger nodes indicate more significant association with certain sport group). Colors represent classes of metabolites (Benzoate metabolites in yellow, chemicals in green, food components in red and xanthine metabolites in blue)

GGM sub-network was constructed for all xenobiotics with no missing values (*n* = 72) with size of nodes reflecting the level of significance, thus creating a map that potentially reveals biochemical connections between different sport groups-associated networks containing ≥2 metabolites (Fig. 3). These included two xanthine sub-networks showing partial correlations among 11 xanthine metabolites (shown in blue in Fig. 3), one benzoate sub-network including 5 benzoate metabolites (shown in yellow in Fig. 3) and various smaller food components sub-networks that revealed direct partial correlations between 4-vinylguaiacol sulfate and ferulic acid 4-sulfate and between methyl glucopyranoside (alpha/beta) and stachydrine (shown in red in Fig. 3).

## Discussion

Athletes’ optimal nutritional demand is dictated by their sport-related energy demand and training regimen as well as athlete’s own metabolic requirement. Maintaining optimal nutritional need would improve performance and recovery from exercise and injury whereas inadequate nutrition could compromise both. Such optimal nutritional need varies among different sports although the most remarkable finding when reviewing the literature is the scarcity of such data [13]. Supplements are frequently used by athletes to compensate for nutritional deficiencies and boost nutritional consumption, aiming to achieve optimal energy demand. However, the effectiveness of supplements and their potential adverse effects remains questionable. Information regarding athletes’ supplement consumption is rather scarce and depends mostly on interviews and surveys.

To study the effect of specific dietary components on human health and performance, several groups have utilized metabolomics. Vitamin E supplementation was shown to influence phospholipid metabolism and induce lysoPC generation, a general pro-inflammatory response [14]. A flavonoid-rich diet triggered changes in 63 plasma metabolites with 70% belonging to lipid and xenobiotic super pathways [15]. Polyphenol soy protein complex supplementation was linked to an enhanced gut-derived phenolic signature and ketogenesis in runners during recovery from a 3-d heavy exertion [16]. Consumption of fruits, such as banana and pears, was shown to improve 75-km cycling performance, reduce fatty acid utilization and oxidation, and enhance the anti-oxidant capacity by increasing unique phenolic production [17]. Pistachio nut ingestion was also associated with improved 75-km cycling time and enhanced post-exercise plasma levels of raffinose, sucrose, and metabolites related to oxidative stress [18]. Further studies have adopted a predictive metabolomics approach by examining the effect of composition of macronutrients consumed immediately post-exercise on the serum metabolic profile in the early recovery phase. These studies have suggested that pro-anabolic processes were favored, with a carbohydrate-protein mix compared to water or carbohydrate consumption [19]. Despite their controlled nature, these studies are limited to the supplement in question and may not reflect what is truly consumed by elite athletes.

This study, therefore, followed a different approach by profiling supplement consumption in elite athletes retrospectively by identifying xenobiotics in their sera collected at two Anti-Doping Laboratories. A number of xenobiotics varied significantly among sport groups, including some that potentially originated from drugs, supplements, food products and other chemical contaminants. Although the exact sources of these metabolites and their potential effect on athletes’ performance remain to be confirmed, this study provides a snapshot of xenobiotics present in different sport groups that may carry certain benefits or inflict harm to these athletes.

### Xenobiotics increased in athletics

Athletics that includes a group of competitive sports such as running, jumping, throwing, and walking showed higher concentrations of two xenobiotics that have potentially originated from food products and/or supplements, namely eugenol sulfate and stachydrine. Eugenol is a potent anti-oxidant found in many plants, herbs and spices especially in clove but is also contained in some supplements that claim blood purification and reduction of risk of gingivitis and heart disease [20]. Stachydrine, a biomarker of citrus fruits consumption [21], was also increased in athletics. It is also contained in some supplements promoting calming and soothing effects and relief from anxiety. It can serve as an osmoprotective compound for the kidneys and has been shown recently to exert anti-inflammatory and anti-oxidative stress effects in animal models [22]. There was a partial correlation revealed by GGM sub-networks between stachydrine and methyl glucopyranoside (alpha/beta), also elevated in athletics, suggesting a similar source, perhaps orange juice as previously shown [23]. Other compounds elevated in athletics included 2, 3-Dihydroxy-isovalerate, a substrate of dihydroxyacid dehydratase known to be sensitive to nitric oxide [24] and 4-hydroxyhippurate, a microbial end-product derived from polyphenol metabolism by the microflora in the intestine.

### Xenobiotics increased in football (soccer)

Footballers have shown higher levels of a number of xenobiotics that potentially originate from food products and/or supplements. These include caffeic acid (3,4-dihydroxycinnamic acid) that is produced by all plants including thyme, sage, spearmint, cinnamon, star anise, black chokeberry, tea and coffee and can also be consumed as a supplement. Caffeic acid-treated animals had enhanced exercise tolerance, lower blood lactate and hepatic oxidation [25]. The derivative of caffeic acid (caffeic acid phenethyl ester) was shown previously to protect against hyperthermal stress induced by prolonged exercise [26]. Caffeic acid has also anti-oxidant properties shown both in vitro and also in vivo [27, 28]. Ferulic acid 4-sulfate, a potent ubiquitous plant anti-oxidant found in high concentration in wheat, rice, peanuts, oranges and apples [29] and in oral supplement form, was also high among footballers. Ferulic acid is a ubiquitous plant component generated from phenylalanine and tyrosine metabolism and is a direct product of caffeic acid in plants. Other xenobiotics with higher levels in footballers included potential drugs such as ectoine, a nasal spray and eye drops, mostly used for the treatment of allergic rhinitis and rhinoconjunctivitis symptoms for relief of nose block and sneezing [30], potentially substantiated through continued grass exposure. Another potential drug-related compound is quinate. Quinine, an alkaloid that is synthesized in plants and the active ingredient of quinate, is a used for treating muscle cramps among footballers [31]. Hippurate, a benzoate metabolite, was also higher among footballers. Hippurate is abundant in fruits and whole grains and has been shown to be associated with reduced risk of metabolic syndrome [32]. From the GGM sub-networks, hippurate levels were partially correlated with other benzoate metabolites including catechol sulfate and O-methylcatechol sulfate, both shown to be also increased in football. Other xenobiotics elevated among footballers included 4-vinylguaiacol sulfate, a flavor additive in beer, also found in partial correlation with ferulic acid as shown by GGM sub-network. 2-furoyl glycine, partially correlated with quinate, was also high in footballers. 1.3.7-trimethylurate, a minor metabolite of caffeine, was also elevated in football.

### Xenobiotics increased in boxing

The boxing group, albeit small (*n* = 17) and mainly composed of females, has shown three elevated xenobiotics in their sera compared to other sport groups. One of these was retinol (vitamin A), a nutritional supplement that has potent anti-oxidant properties and is used as an anti-aging cream [33]. Another xenobiotic increased in boxing was 2.pyrrolidinone, the simplest γ-lactam with nootropic effects, providing neuroprotection after stroke and proving efficacious as an antiepileptic agent [34]. The presence of this drug among boxers may reflect a prophylactic treatment for multiple head injury. Thioproline, an intracellular sulfhydril antioxidant and free radical scavenger that enhances immune functions, was also found to be elevated in boxers. Data from in vivo studies in mice showed that thioproline induces an anorexic effect associated with better survival and improved neurological function through a decreased oxidative damage [35].

### Study limitations

Although this is the first study analyzing xenobiotics present in elite athletes from different sports, the lack of important information about participants such as age, body mass index and dietary and training regiments has hindered data interpretation. Additionally, the over representation of one group (footballers) compared to other participating groups may have introduced some bias in the study design that may have influenced the results. Furthermore, a batch effect may have also influenced the data, as athletes’ samples were collected and processed at multiple sites, although a batch correction was applied as described in the methods section. Another potential limitation of this study is the lack of information related to the precise role of each athlete in their team, which could impact their energy and endurance requirement [36]. However, the large number of participants and their wide range of sport groups may have diluted out the effects of some of these potential confounders that were unaccounted for in our statistical model.

Assessment of the impact of changes in the dietary nutrient content on metabolic profiles is rather complicated as it overlaps with non-nutrient signals absorbed through environmental exposure. Both nutrient and non-nutrient contents are further processed by the gut’s microflora, thereby producing significant metabolic signals and adding to the complexity of the metabolome of biofluids in human nutrition [9]. Therefore, recommendations on best practices when performing human intervention studies such as integration of OMICS data (including microbiome) with habitual dietary and lifestyle information (standardized FFQ) were recently suggested for better data interpretability [37, 38].

## Conclusion

Athletes from different sports may use different dietary supplements depending on the nature of the physical activities they perform and the desired outcomes from the dietary supplements, aiming ultimately for better performance and recovery from exercise and injury. This paper provides evidence that athletes from different sports exhibit a distinct xenobiotic profile that may reflect their drug/supplement use, diet and exposure to various chemicals. These include metabolites that potentially prolong exercise tolerance, provide a nootropic effect, exert a potent anti-oxidant effect or originate from drugs for different types of injuries. GGM provided metabolic networks that linked various xenobiotics associated with different sport groups, offering further evidence of potentially functional relationship among these xenobiotics. Replication studies and administration trials that adopt integrated OMICS approach data with dietary and lifestyle information are warranted to confirm differences in the metabolic profiles associated with different sport groups in independent data sets, aiming ultimately for better assessment of supplement use by athletes and monitoring of their exposure to various environmental chemicals that may provide benefit or pose harm to these athletes.

## Abbreviations

ESI: Electrospray ionization
FA: Formic acid
GGMs: Gaussian Graphical Models
HESI-II: Heated electrospray ionization
KEGG: Kyoto Encyclopedia of Genes and Genomes
LIMS: Laboratory Information Management System
OPLS-DA: Orthogonal partial least square discriminant analysis
PFPA: Perfluoropentanoic acid
QC: Quality control
RI: Retention time/index
RSD: Relative standard deviation
UPLC: Ultra-performance liquid chromatography
UPLC-MS/MS: Ultrahigh Performance Liquid Chromatography-Tandem Mass Spectroscopy
